# Drug Retention Rate and Predictive Factors of Drug Survival for Interleukin-1 Inhibitors in Systemic Juvenile Idiopathic Arthritis

**DOI:** 10.3389/fphar.2018.01526

**Published:** 2019-01-08

**Authors:** Jurgen Sota, Antonella Insalaco, Rolando Cimaz, Maria Alessio, Marco Cattalini, Romina Gallizzi, Maria Cristina Maggio, Giuseppe Lopalco, Francesco La Torre, Claudia Fabiani, Manuela Pardeo, Alma Nunzia Olivieri, Paolo Sfriso, Carlo Salvarani, Carla Gaggiano, Salvatore Grosso, Claudia Bracaglia, Fabrizio De Benedetti, Donato Rigante, Luca Cantarini

**Affiliations:** ^1^Research Center of Systemic Autoinflammatory Diseases and Behçet’s Disease Clinic, Department of Medical Sciences, Surgery and Neurosciences, University of Siena, Siena, Italy; ^2^Division of Rheumatology, Department of Pediatric Medicine, Bambino Gesù Children’s Hospital, IRCCS, Rome, Italy; ^3^Rheumatology Unit, Meyer Children’s Hospital, University of Florence, Florence, Italy; ^4^Department of Pediatrics, University of Naples Federico II, Naples, Italy; ^5^Pediatric Clinic, University of Brescia, Brescia, Italy; ^6^Department of Pediatrics, Azienda Ospedaliera Universitaria Policlinico “G. Martino” – Messina, University of Messina, Messina, Italy; ^7^Universitary Department “Pro.S.A.M.I.”, University of Palermo, Palermo, Italy; ^8^Rheumatology Unit, Department of Emergency and Organ Transplantation, University of Bari, Bari, Italy; ^9^Pediatric Rheumatology Section, Pediatric Oncoematology Unit, Vito Fazzi Hospital, Lecce, Italy; ^10^Ophthalmology Unit, Department of Medicine, Surgery and Neuroscience, University of Siena, Siena, Italy; ^11^Dipartimento della Donna, del Bambino e di Chirurgia Generale e Specialistica, Seconda Università degli Studi di Napoli, Naples, Italy; ^12^Rheumatology Unit, Department of Medicine, University of Padua, Padua, Italy; ^13^Rheumatology Unit, Department of Internal Medicine, Azienda Ospedaliera ASMN, Istituto di Ricovero e Cura a Carattere Scientifico, Reggio Emilia, Italy; ^14^Clinical Pediatrics, Department of Molecular Medicine and Development, University of Siena, Siena, Italy; ^15^Institute of Pediatrics, Periodic Fever Research Center, Università Cattolica Sacro Cuore, Fondazione Policlinico A. Gemelli, IRCCS, Rome, Italy

**Keywords:** systemic juvenile idiopathic arthritis, interleukin 1-beta, therapy, anakinra, canakinumab, drug retention rate

## Abstract

**Background and Objectives:** Few studies have reported the drug retention rate (DRR) of biologic drugs in juvenile idiopathic arthritis (JIA), and none of them has specifically investigated the DRR of interleukin (IL)-1 inhibitors on systemic JIA (sJIA). This study aims to describe IL-1 inhibitors DRR and evaluate predictive factors of drug survival based on data from a real-world setting concerning sJIA.

**Methods:** Medical records from sJIA patients treated with anakinra (ANA) and canakinumab (CAN) were retrospectively analyzed from 15 Italian tertiary referral centers.

**Results:** Seventy seven patients were enrolled for a total of 86 treatment courses. The cumulative retention rate of the IL-1 inhibitors at 12-, 24-, 48-, and 60-months of follow-up was 79.9, 59.5, 53.5, and 53.5%, respectively, without any statistically significant differences between ANA and CAN (*p* = 0.056), and between patients treated in monotherapy compared to the subgroup co-administered with conventional immunosuppressors (*p* = 0.058). On the contrary, significant differences were found between biologic-naive patients and those previously treated with biologic drugs (*p* = 0.038) and when distinguishing according to adverse events (AEs) occurrence (*p* = 0.04). In regression analysis, patients pre-treated with other biologics (HR = 3.357 [CI: 1.341–8.406], *p* = 0.01) and those experiencing AEs (HR = 2.970 [CI: 1.186–7.435], *p* = 0.020) were associated with a higher hazard ratio of IL-1 inhibitors withdrawal. The mean treatment delay was significantly higher among patients discontinuing IL-1 inhibitors (*p* = 0.0002).

**Conclusions:** Our findings suggest an excellent overall DRR for both ANA and CAN that might be further augmented by paying attention to AEs and employing these agents as first-line biologics in an early disease phase.

## Introduction

Systemic onset-juvenile idiopathic arthritis (sJIA) is the most severe and distinct category of JIA due to its unique pathogenesis, severity and disproportionately high morbidity and mortality rates when compared to other JIA subtypes (Ruperto et al., 2012; Minoia et al., 2014; Kumar, 2016; Davies et al., 2017). This condition is distinguished by its unique clinical features and treatment responses, that make it similar to the autoinflammatory diseases, a large family of pathologic entities caused by dysregulation of the innate immune system leading to recurrent or continuous inflammation (Rigante, 2017, 2018). Treatment of sJIA is often challenging and, when long-term administration of corticosteroids (CS) is needed, conventional disease modifying anti-rheumatic drugs (cDMARDs) or biologic drugs should be introduced to replace or at least reduce CS daily intake. Furthermore, patients affected by sJIA are often recalcitrant to traditional/standard therapies (Cimaz, 2016; Mauro et al., 2017). Recent genetic and immunologic studies have disclosed the emerging role of interleukin (IL)-1 and genetic polymorphisms in promoter elements and genes of IL-1 family into sJIA pathogenesis, reporting an altered innate immune response with overproduction of IL-1, IL-6, and IL-18 (Mellins et al., 2011). This has been translated into clinical practice via cytokine-targeted treatments, particularly with IL-1 inhibitors after showing their paramount clinical efficacy in patients with cryopyrin-associated periodic syndrome (Cantarini et al., 2011). The advent of biologic agents has revolutionized therapeutic approaches in sJIA as their introduction has been shown to modify disease course and improve overall outcomes, particularly when initiated early (Nigrovic et al., 2011; Hedrich et al., 2012; Vastert et al., 2014; Pardeo et al., 2015). Several studies also including randomized clinical trials display promising results of IL-1 blocking agents in the management of this severe disease (Quartier et al., 2011; Ruperto et al., 2012; Vastert et al., 2014; Feist et al., 2018).

However, while robust evidence from randomized clinical trials is available in the medical literature, only a small amount of real-life data has been published so far, especially in the context of early treatment, and few of them have marginally investigated anti-IL-1 agents drug retention rate (DRR) on a relatively long-term follow-up. On this basis, we herein report our multicenter experience on a patients affected by sJIA treated with the anti-IL-1 agents anakinra (ANA) and canakinumab (CAN), focusing on their long-term effectiveness and predictive factors of discontinuation.

## Materials and Methods

### Study Design and Participants

Medical records of 76 patients affected by sJIA enrolled from January 2008 until July 2016 in 15 Italian tertiary referral centers were retrospectively reviewed. The following demographic, clinical, and therapeutic data were collected: age, gender, age at disease onset, disease duration, treatment delay, the anti-IL-1 agent employed, dosages used, concomitant and previous treatments, anti-IL 1 treatment duration, and reasons for discontinuation.

All patients were diagnosed according to the revised International League of Association for Rheumatology (ILAR) criteria (Petty et al., 2004). In accordance with the best standards of care, they were systematically followed-up every 3 months and/or in case of necessity (disease flare and/or safety issues). Before starting anti-IL-1 treatment with ANA or CAN, patients underwent a complete medical examination, evaluation of hepatitis B and hepatitis C virus markers, urine culture, QuantiFERON test and chest X-ray to rule out active or latent infections. ANA and CAN dosages ranged from 1 to 4 mg/kg and from 2 mg/kg every 8 weeks to 4 mg/kg every 4 weeks, respectively.

### Protocol Approval

This study adhered to the tenets of the Declaration of Helsinki and the protocol was approved by the local Ethic Committee (reference number: 364-16OCT2013). Written informed consent was obtained from all patients’ legal guardians.

### Aims and Endpoints

The primary aim of the study was to examine the overall DRR of IL-1 blockers in sJIA patients. Secondary aims of our study were to: (i) explore the influence of biologic line of treatment, adverse events (AEs), type of anti-IL-1 agent, and the concomitant use of cDMARDs on DRR; (ii) find eventual predictive factors associated with events leading to drug discontinuation. The CS sparing effect and the impact of disease duration and treatment delay on survival constituted ancillary aims. The primary endpoint was evaluated by Kaplan–Meier survival curve at 12-, 24-, -48, and 60-months of follow-up. Secondary endopoints were as follows: (i) using limit estimators to compare survival curves according to AEs, ANA vs. CAN, monotherapy vs. combination therapy with cDMARDs and significant differences on survival curves distinguishing between biologic-naive patients and those already treated with other biologics; (ii) to evaluate whether demographic, clinical, and therapeutic variables could predict time to treatment discontinuation. Finally, the ancillary aims were explored by any potential statistically significant differences in the mean disease duration and in the mean treatment delay, subdividing our sample in patients continuing and those discontinuing the treatment as well as on CS-sparing effect.

### Statistical Analysis

Data were analyzed using IBMSPSS Statistics for Windows, version 24 (IBM Corp., Armonk, NY, United States). Descriptive statistics was employed to display mean and standard deviation (SD) or median and interquartile range (IQR) as appropriate. Shapiro–Wilk test is the test by which we assessed the normality of our data. Categorical variables were analyzed by Pearson’s chi-square test, and McNemar test for repeated measures, while differences in means were investigated with Mann–Whitney *U* test. Survival analysis were carried out with Kaplan–Meier curves with the event being ANA or CAN discontinuation. Patients discontinuing treatment due to remission were not included in the statistical analysis. Survival curves were compared using both long-rank and Breslow test as limit estimators. Event-free survival was assessed with a Cox proportional hazard model using 95% confidence interval for hazard ratios aiming to evaluate any relation of demographic, clinical and therapeutic data with DRR. The threshold for statistical significance was set to *p* < 0.05 and all *p-*values were two-sided.

## Results

We studied 86 treatment regimens administered in a cohort of 77 pediatric patients (34 males, 43 females) receiving anti-IL-1 agents from January 2008 to July 2016. Demographic, clinical and therapeutic data are shown in Table 1. ANA and CAN were used in 61 and 25 regimens, respectively. The mean ± SD time of treatment duration was 22.67 ± 19.50 months. The median age ± IQR at disease onset was 6.00 ± 7.10 years.

**Table 1:** Demographic, clinical and therapeutic data of our cohort of patients affected with sJIA.

Patients no.	77
Male/female no.	34/43
Mean age ± SD	12.71 ± 6.65
Age at onset (median ± IQR)	5.65 ± 7.40
Age at diagnosis (median ± IQR)	5.75 ± 7.48
Disease duration (median ± IQR)	4.00 ± 5.95
Treatment delay (median ± IQR)	2.33 ± 6.31
Previous biologics	ETN (*n* = 13); IFX (*n* = 3); ADA (*n* = 4); TCZ (*n* = 5); ABA (*n* = 3); RTX (*n* = 2); GOL (*n* = 2); CZP (*n* = 1)
Concomitant cDMARDs	MTX (*n* = 12); CsA (*n* = 11); SSZ (*n* = 1); LFN (*n* = 1); HCQ (*n* = 1)


*ABA, abatacept; ADA, adalimumab; cDMARDs, conventional disease modifying anti-rheumatic drugs; CsA, cyclosporine; CZP, certolizumab; ETN, etanercept; GOL, golimumab; HCQ, hydroxychloroquine; IFX, infliximab; IQR, interquartile range; LFN, leflunomide; MTX, methotrexate; RTX, rituximab; SD, standard deviation; sJIA, systemic-onset juvenile idiopathic arthritis; SSZ, sulfasalazine; TCZ, tocilizumab*.

**FIGURE 1:**
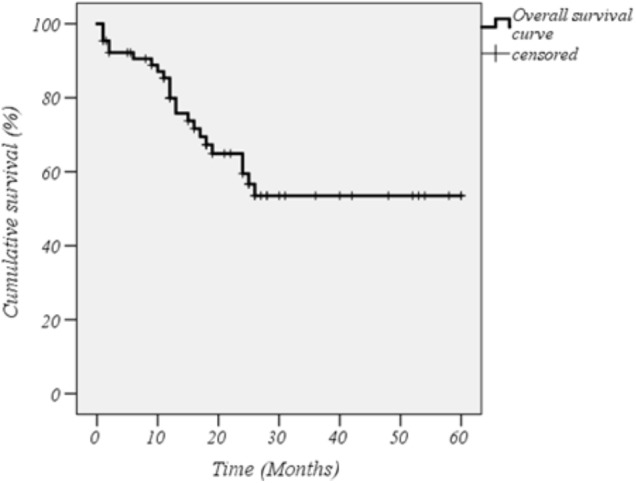
Kaplan–Meier curve related to the overall cumulative drug retention rate of interleukin-1 inhibitors in the whole cohort of patients with systemic onset-juvenile idiopathic arthritis. Time 0 represents the beginning of therapy with the event being the treatment discontinuation.

**FIGURE 2:**
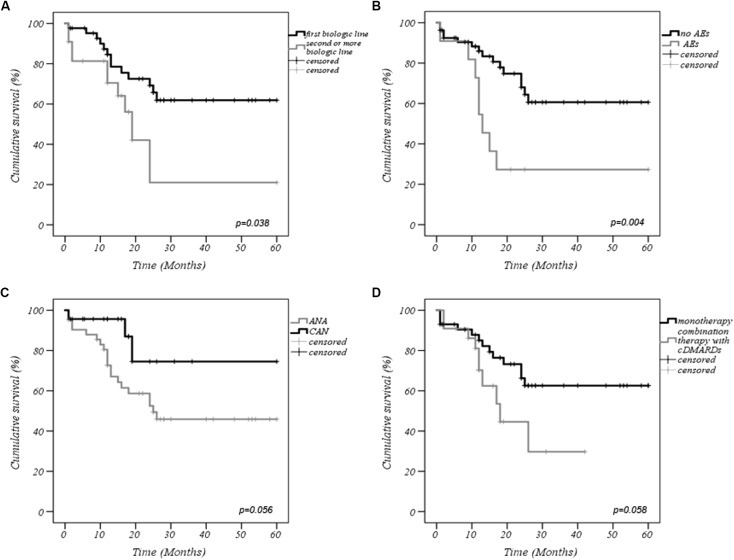
Kaplan–Meier curves describing the cumulative survival of interleukin-1 (IL-1) inhibitors according to: **(A)** biologic line of treatment, **(B)** occurrence of AEs, **(C)** type of anti-IL-1 agent employed, **(D)** concomitant use cDMARDs or monotherapy with biologic agents. AEs, adverse events; ANA, anakinra; CAN, canakinumab; cDMARDs, conventional disease modifying anti-rheumatic drugs.

The cumulative retention rate of both anti-IL-1 agents at 12-, 24-, 48-, and 60-months of follow-up was 79.9, 59.5, 53.5, and 53.5%, respectively (Figure 1). Twenty-two out of 77 patients were co-administered with cDMARDs, and 22 subjects had been previously exposed to other biologic drugs. Statistically significant differences were observed between biologic-naive patients and those previously treated with biologic drugs (*p* = 0.038) (Figure 2A). As illustrated in Figure 2B, the DRR of IL-1 blockers resulted significantly different when separating the cohort according to the occurrence of AEs (Breslow test *p* = 0.006 and log-rank *p* = 0.004). Contrarily, no statistically significant differences were detected between ANA and CAN (*p* = 0.056) (Figure 2C), and between patients treated in monotherapy compared to the subgroup co-administered with cDMARDs (*p* = 0.058) (Figure 2D), while Cox regression analysis identified two variables associated with a higher hazard of treatment withdrawal: biologic line, with a higher hazard for biologic-exposed patients (HR = 3.357 [CI: 1.341–8.406], *p* = 0.01) and the occurrence of AEs (HR = 2.970 [CI: 1.186–7.435], *p* = 0.020).

Additionally, the median disease duration was significantly higher among patients discontinuing IL-1 blockers (5.88 years ± 6.55) compared to the subgroup that was able to retain these biologic agents (3.17 years ± 3.68) (*p* = 0.011). This difference was also found with regard to treatment delay, which was significantly higher in the subgroup of patients discontinuing anti-IL-1 agents (3.71 years ± 6.07) vs. (1.18 years ± 2.53) of those still on treatment (*p* = 0.0002).

With regard to the CS-sparing effect, a significant reduction in the number of patients requiring the support of CS was found (*p* = 0.025). Sixteen out of 63 patients (27%) were able to completely discontinue CS therapy.

**FIGURE 3:**
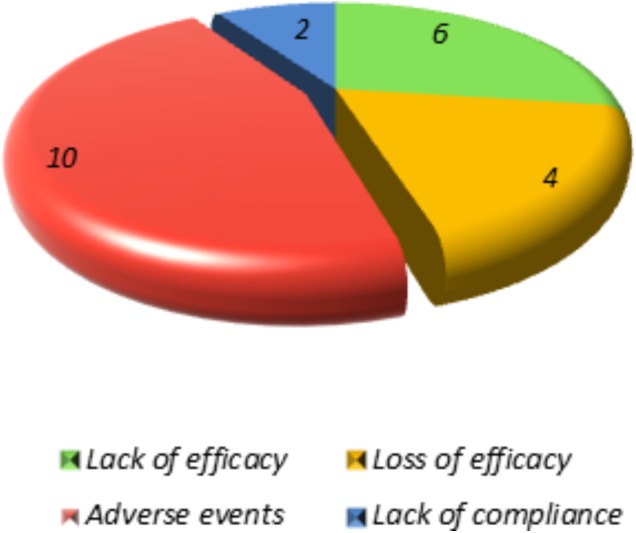
Reasons for discontinuation in sJIA patients.

The AEs occurred in 13 out of 77 patients (17.1%) (11 on ANA and 2 on CAN), with the most frequent being injection site-reactions (*n* = 7), followed by generalized skin rashes (*n* = 4), respiratory problems (*n* = 1), and abnormal level of liver enzymes (*n* = 1). Two patients exhibiting generalized skin rash presented also diarrhea. Overall, AEs were responsible for 10 cases of treatment discontinuation. Figure 3 shows the reasons for treatment discontinuation. No serious AEs were recorded, and none of our patients developed macrophage activation syndrome during the follow-up period.

## Discussion

Among the JIA spectrum, the systemic category represents a difficult-to-manage condition not only from a diagnostic perspective, due to the absence of pathognomonic clinical features or laboratory markers forcing the clinician to perform a thorough diagnostic work-up, but also from a therapeutic viewpoint. In the pre-biologic era, CS have been the sole effective treatment option for many years, and the mainstay of therapy in most patients with sJIA, while cDMARDs and some newer alternatives such as anti-tumor necrosis factor-α agents had provided unsatisfactory results (Russo and Katsicas, 2009; Stoll and Cron, 2014). Pathogenetic evidence suggesting a prominent role of IL-1 in sJIA pathways has given the rationale for specific cytokine-targeted agents (Mellins et al., 2011). From the first observation of a successful treatment of sJIA with IL-1 inhibition in 2004 (Verbsky and White, 2004), a growing body of evidences confirming the efficacy of anti-IL-1 agents has matured over time (Lequerré et al., 2008; Quartier et al., 2011; Ruperto et al., 2012; Vastert et al., 2014; Pardeo et al., 2015; Feist et al., 2018).

In the present study we have reported the real-life experience of 15 Italian tertiary referral centers with IL-1 inhibitors used in the treatment of sJIA, focusing on long-term effectiveness expressed as DRR.

Our findings suggest an excellent DRR with an estimated probability greater than 50% to continue the treatment after 5 years from the start. The DRR tends to decrease in the 1st years of treatment and subsequently stabilizes: this may be at least partially explained by the higher risk of AEs during the initial period, which decreases over time from the initiation of IL-1 inhibition, as reported in a previous paper investigating safety profile of IL-1 blockade (Sota et al., 2018). Indeed, safety issues determined a significant decrease in DRR not only on the long-term, but also in the beginning of anti-IL-1 therapy, as shown in the survival curves.

Nonetheless, difficulties were encountered in comparing our results with the available medical literature due to different outcome measures and biologics administered as well as heterogeneity of populations enrolled in the other studies without a specific investigation on sJIA. Only a few studies have investigated DRR in JIA patients (Tynjälä et al., 2009; Otten et al., 2013). Otten et al. (2013) reported 17 cases of sJIA treated with second or third line ANA, which presented a superior DRR compared to a second TNF-α blocker and reported that the effectiveness of a second biologic agent seemed low, especially in the case of discontinuation of the primary biologic agent due to inefficacy. In line with this notion, our findings suggest a better DRR of IL-1 inhibitors when used in biologic-naive patients. Other authors have also recommended to administer IL-1 inhibitors as first-line agents instead of a rescue therapy (Nigrovic et al., 2011; Hedrich et al., 2012; Vastert et al., 2014; Pardeo et al., 2015), which is also contemplated in the new ACR recommendations for the treatment of sJIA (Ringold et al., 2013). Another relevant issue concerns the need for a timely introduction of modern cytokine-blocking strategies to prevent or at least minimize structural damage due to long-term disease and treatment-related AEs. Our data highlight the significant impact of treatment delay in DRR of IL-1 inhibitors. Indeed, early IL-1 blockade may take advantage of the “window of opportunity” and modify disease evolution (Nigrovic, 2014), while avoiding long-term sequelae which have proven to be detrimental in a limited-resource setting where early diagnosis or access to multidisciplinary care and to biologics are still obstacles to overcome (Dewoolkar et al., 2017). A more chronic and persistent disease course has been associated with a delay in diagnosis and consequently in starting an appropriate treatment. On the other hand, earlier treatment has been associated with better outcomes (Pardeo et al., 2015; Dewoolkar et al., 2017). This may be attributable, as suggested by several authors, to the unique pathogenesis of sJIA, which has been hypothesized to be predominantly autoinflammatory in its early stages explaining the better outcome for IL-1 blockade in this phase (Nigrovic, 2014; Pardeo et al., 2017). Timely treatment is imperative also for minimizing CS exposure and CS-related deleterious AEs. In particular, we found a significant lower number of patients on CS at the last follow-up visit, which is of crucial importance in the pediatric age.

We observed an excellent safety profile of ANA and CAN, that were well-tolerated without any serious AEs and no case of macrophage activation syndrome.

Limitations of our study includes the not randomized retrospective design along with its inherent associated drawbacks, and the difficulties for a valid comparison with the available literature due to different endpoints. Although the vast majority of patients were treated with standard dosages, a few subjects were administered with higher dosages. However, the small sample size of patients administered with non-standard dosages did not allow to perform a reliable statistical analysis. In addition, the decision on when to start the treatment was made by the local physician without any predefined criteria. To the best of our knowledge, this is the first study to report the long-term effectiveness in terms of DRR of anti-IL-1 agents in a cohort of patients diagnosed with sJIA.

## Conclusion

Despite remaining a major therapeutic challenge in pediatric rheumatology, sJIA may be currently managed with new biotechnologic drugs, including anti-IL-1 agents, which allow to improve long-term outcome as well as to minimize long-term exposure to CS. Indeed, our results have shown an excellent overall retention rate of the IL-1 inhibitors ANA and CAN. Both IL-1 inhibitors showed a similar DRR, and their survival was not affected by the concomitant use of cDMARDs. These data highlight the effectiveness of these agents in sJIA patients as monotherapy. A close attention on safety concerns is warranted, especially in the 1st year after treatment initiation. On the other hand, the retention rate of anti IL-1 agents was influenced by the different line of biologic therapy, suggesting that ANA and CAN should be used as first-line biologics. Finally, our results have displayed a significant impact of treatment delay in drug discontinuation. Indeed, early treatment may take advantage of the “windows of opportunity,” but this hypothesis is far from being proven and requires further studies for its corroboration.

## Author Contributions

JS and LC conceived and designed the study. JS performed the statistical analysis. JS and LC wrote the first draft of the manuscript. DR revised the overall data and revised the manuscript. All authors critically reviewed the draft manuscript and approved the submitted version.

## Conflict of Interest Statement

The authors declare that the research was conducted in the absence of any commercial or financial relationships that could be construed as a potential conflict of interest.
